# High burden of hypertension amongst adult population in rural districts of Northwest Ethiopia: A call for community based intervention

**DOI:** 10.1371/journal.pone.0275830

**Published:** 2022-10-13

**Authors:** Destaw Fetene Teshome, Shitaye Alemu Balcha, Tadesse Awoke Ayele, Asmamaw Atnafu, Mekonnen Sisay, Marye Getnet Asfaw, Getnet Mitike, Kassahun Alemu Gelaye

**Affiliations:** 1 Department of Epidemiology and Biostatistics, Institute of Public Health, College of Medicine and Health Sciences, University of Gondar, Gondar, Ethiopia; 2 Department of Internal Medicine, School of Medicine, College of Medicine and Health Sciences, University of Gondar, Gondar, Ethiopia; 3 Department of Health Systems and Policy, Institute of Public Health, College of Medicine and Health Sciences, University of Gondar, Gondar, Ethiopia; 4 Department of Human Nutrition, Institute of Public Health, College of Medicine and Health Sciences, University of Gondar, Gondar, Ethiopia; 5 Department of Emergency Nursing, School of Nursing, College of Medicine and Health Sciences, University of Gondar, Gondar, Ethiopia; 6 International Institute for Primary Health Care-Ethiopia, Addis Ababa, Ethiopia; Kasturba Medical College, Manipal Academy of Higher Education, Manipal, INDIA

## Abstract

**Background:**

Hypertension is a serious public health issue in Ethiopia, but there is a paucity of evidence in the country’s rural areas. The aim of this study was to determine the prevalence of hypertension and its risk factors among adults in rural districts in northwest Ethiopia.

**Methods:**

A community-based cross-sectional study was conducted from June to October 2020. The 1177 study participants were chosen using a multistage sampling procedure. A face-to-face interview was conducted using an adapted version of the WHO STEPwise approach questionnaire. Blood pressure was measured three times using an aneroid sphygmomanometer, and the mean of the last two readings were used for the analysis. Data was entered using Epidata and analyzed using STATA-16. Multivariable logistic regression was used to identify risk factors associated with hypertension.

**Results:**

Of the total participants, 218 (18.5%) were found to be hypertensive. The prevalence of hypertension consistently increases with age. Hypertension was positively and significantly associated with female sex ((adjusted odd ratio (AOR) = 2.30, 95% CI: 1.53, 3.45)), age group 45–54 years (AOR = 4.63, 95% CI: 1.01, 21.37), 55–64 years (AOR = 14.40, 95% CI: 3.07, 67.63), ≥65 years (AOR = 19.37, 95% CI: 4.03, 93.09), having history of alcohol consumption (AOR = 3.25, 95% CI: 1.17, 9.02), used much amount of salt (AOR = 2.37, 95% CI: 1.53, 3.60) and too much amount of salt (AOR = 3.78, 95% CI: 1.85, 7.72), sleeping for a short duration (AOR = 2.05, 95%CI: 1.30, 3.24), and having family history of hypertension (AOR = 2.12, 95% CI; 1.32, 3.39).

**Conclusions:**

Hypertension was significantly high among the rural population we studied and is emerging as a public health problem. Female sex, advanced age, ever used alcohol, excessive salt intake, insufficient sleep, and a family history of hypertension were factors that were positively and significantly associated with hypertension. We recommend local health authorities integrate promotion of hypertension health education, lifestyle modification intervention on salt and alcohol reduction, and hypertension detection, particularly for the female and elderly population, at the health post level to avert the problem.

## Introduction

Hypertension, commonly known as high blood pressure, is a chronic medical disorder in which the blood pressure (BP) in the arteries remains high for an extended period of time [1]. It is a major public health issue that affects people all around the world. In 2016, an estimated 1.13 billion adults worldwide had hypertension, with two-thirds of them living in low and middle-income countries [2]. The World Health Organization (WHO) African region has the highest rate of hypertension (27%) in the world [3]. It rises as high as 40% and 46% among people age ≥18years [4] and >25 years [5], respectively, in some African settings.

Hypertension is the most important modifiable risk factor for coronary heart disease, stroke, and other cardiovascular diseases (CVDs), accounting for half of global CVDs morbidity and mortality [6]. It is thought to be responsible for about 45% of heart disease deaths, 51% of stroke deaths [7–9], and 18% of global deaths each year [10]. Hypertension complications necessitate costly therapies such as cardiac bypass surgery, carotid artery surgery, and dialysis, all of which drain both individual and government budgets [8, 11].

Noncommunicable diseases, which account for 39% of all deaths in Ethiopia, have become a public health problem [12]. Another report also found that noncommunicable diseases, including CVDs, are responsible for about 37.5% of the disease burden and 43.5% of the deaths in the country [13]. Hypertension has now become a public health issue in Ethiopia, accounting for 62.3% of CVDs [14], 36.3% to 69.3% of stroke cases [15–19], and 3.5% of all the deaths [20]. In Ethiopia, hypertension affects more than 20% of the adult population [21]. Several community based studies have been conducted in Ethiopian cities and revealed a high prevalence of hypertension. For instance, the prevalence of hypertension ranged from 25.1% to 31.9% in the Amhara region of northwest Ethiopia [22–26], 19.7% to 35.2% in Southern Ethiopia [27–30], and 25% to 32.3% in Addis Ababa [31–34].

There is, however, limited evidence on the prevalence and risk factors of hypertension in rural Ethiopia, where the majority of the population (96.2%) eats too much salt (≥5grams per day) [13], a known modifiable risk factor for hypertension [35–37]. Hence, the aim of this study was to determine the prevalence of hypertension and what variables contribute to it in people aged ≥18 years. We analyzed the data from the study “improving hypertension management through task sharing with the Health Extension workers in rural districts of northwest Ethiopia”. Generating such evidence is of paramount importance to develop effective strategies for the prevention and control of hypertension.

## Materials and methods

### Study design and setting

A community based cross sectional study design was conducted in rural areas Dabat and Gondar Zuria districts of northwest Ethiopia from June to October, 2020. Dabat and Gondar Zuria districts are located in the Amhara regional state’s North Gondar and Central Gondar Zones, respectively. Rain-fed agriculture is the main source of income in Dabat and Gondar Zuria districts. Agriculture in the area is characterized by a subsistence mixed farming system that includes livestock production. Almost all cropland is dedicated to annual food crops such as cereals (maize, barley, and teff), oil seeds, and vegetables (sesame) [38]. The study settings were previously described in detail [39].

### Study population and sampling

The sample size was determined using a single population proportion formula with a 14.7% estimated prevalence of hypertension in rural areas [40], 95% confidence interval, 3% margin of error, design effect of 2, and a 10% non-response rate. The study participants were chosen using a multi-stage sampling strategy. First, using a simple random sampling technique, 20 of the total rural kebeles in the two districts were chosen. Second, 3–4 villages have chosen using a simple random sampling technique. Finally, one participant for the study was chosen at random from each household. The sampling strategy was described in detail elsewhere [39]. All adult population age ≥18 years who had lived in the study area for at least 6 months were eligible, however pregnant women were excluded because pregnancy induced hypertension could lead to an overestimation of the true prevalence.

### Data collection tools, procedures and measurements

The data was collected using a structured questionnaire adapted from the WHO STEP-Wise approach to noncommunicable disease surveillance, which included socio-demographic characteristics, behavioral and lifestyle factors, psychosocial stress levels, family history of hypertension, and comorbidities [41]. The questionnaire was first prepared in English and then translated into the local language (Amharic) by two bilingual translators. The other two independent bilingual translators re-translated the translated Amharic version back to the source language to ensure compliance with the original version of the questionnaire.

This study was conducted as part of the research project “improving hypertension management through task sharing with the Health Extension workers in rural districts of northwest Ethiopia”. Three days of theoretical and practical training were provided to Health Extension workers, data collectors (health professionals having MSc in emergency medicine and critical care nursing and Public Health officers), and supervisors. The theoretical session covered hypertensive disease definition, symptoms, main risk factors, complications, prevention methods, and blood pressure measurements. The practical session covered precautions to take before taking BP measurements, proper body positioning during measurement, and how to take BP measurements.

The Health Extension workers, with trained data collectors, visited residents’ homes and provided health information on hypertensive disease and the importance of hypertension screening to study participants. They asked participants if they drank caffeinated beverages like coffee or tea, and worked within 30 minutes. Before their blood pressure was measured, the participants were allowed to sit quietly for 5 minutes. Participants in the study were instructed to sit with their back straight, feet flat on the floor, legs uncrossed, and arm supported on the knees with the upper arm at heart level. To conduct a reliability test with the trained data collectors, the Health Extension workers took the initial BP reading in the sitting position at the left arm using an aneroid sphygmomanometer with an appropriate size brachial pressure cuff and stethoscope. The Health Extension workers palpated the brachial artery of the participants and put the stethoscope bell over it. They inflated the BP cuff while listening to the pulse sounds with the stethoscope until no sounds were heard. They slowly deflated the BP cuff by 2 mmHg per second while listening for the systolic and diastolic readings. The data was collected by five trained data collectors using an interviewer-administered questionnaire. The data collectors also independently took two BP readings for the same participant, one before and one after the interview, 30 minutes apart. To confirm the diagnosis of hypertension, the average of the last two measurements was taken.

Individuals’ weight was measured using a calibrated weight measuring scale. Participants were asked to wear light clothing, and their weight was recorded using a digital scale to the nearest 0.1 kg. The participants’ heights were measured with a tape to the nearest 0.1 cm. Participants were instructed to stand upright, without shoes, with their heels together, and their gaze directed forward. The body mass index (BMI) was calculated using the formula weight in kg/height in m^2^ and classified as underweight (<18.5), normal weight (18.5–24.9), overweight (25–29.9), or obese (≥ 30) [42]. The wealth of the families was calculated using the household assets of the rural community, such as the type of flooring, roof, and walls; the number of rooms in the house; land ownership for agriculture and total amount of agricultural products; livestock ownership, bank account, and solar light source [43]. Based on their relative position on the household wealth index, these were combined into a single wealth index and then divided into three equal-sized groups (poor, medium, and reach).

On a regular basis, the dial sphygmomanometers were calibrated against a standard mercury sphygmomanometer to ensure consistency and accuracy of the readings. Information exchange by telephone and close supervision by the principal investigator and supervisor were made on a daily basis. Coding and data cleaning have done.

### Operational definitions

At the time of screening, hypertension is defined as a systolic blood pressure of ≥140 mmHg or a diastolic blood pressure of ≥90 mmHg or being on antihypertensive medication [44]. Alcohol intake was assessed by asking participants if they had ever consumed alcohol and categorized them as alcohol users if they used alcohol regularly or occasionally. Any combinations of walking, moderate or vigorous intensity activity with an average metabolic equivalents-minutes per week of <600, 600–2999 and ≥3000 were used to classify the physical activity of the participants as low, moderate, and high level, respectively [45]. The question, “how many hours do you sleep a day?” was used to determine sleep duration [46]. A sleep duration of less than 6 hours per 24 hours was considered as short sleep duration [47]. A WHO STEPwise approach tool of self-reported quantity of salt consumption using the question "how much salt do you think you consume?" was used to assess the perception of the amount of salt they consumed. Self-reports of much or too much salt consumption in processed foods, adding salt when cooking, and/or to cooked meals were used to define participants’ much and too much of salt consumption [41].

### Data processing and analysis

Epidata version 4.6 was used to enter data, which was then exported to STATA version 16 for further analysis. The data were checked for missing values, cleaned for completeness, recoded, and computed for variables that could be calculated. The results were reported using frequency, percentages, means with standard deviations, and median with interquartile range (IQR). The data was also presented via tables and figures. Bivariable and multivariable binary logistic regression analysis were both carried out. Variables that have significant associations with hypertension during multivariable analysis were identified using adjusted odds ratios with 95% CI and p-values of <0.05. The model fitness of good test was also computed and reported.

### Ethical approval and consent to participate

This study was approved by the Institutional review board of the University of Gondar (Ref. No: V/P/RCS/05/1580/2020). An information sheet was prepared that included the study’s purpose and significance, benefits and risks, the confidentiality of the participants’ responses and explained to study participants. Because the study was conducted in rural settings and the majority of the study participants could not read and write, verbal informed consent was obtained from them. The Institutional review board of the University of Gondar gave their approval to use verbal informed consent. Participant involvement in the study was based on a voluntary basis. The opportunity to ask any question about the study, as well as the right to refuse or terminate the interview, was provided. Data confidentiality was ensured by using identification numbers rather than names and restricting access to the data.

## Results

### Participants’ socio-demographic characteristics

A total of 1177 adult population participated in the study, resulting in a 100% response rate. The median age of the participants was 41 (IQR = 30–55) years. Of the total participants, 640 (54.4%) were female, 1167 (99.2%) were orthodox Christians, 955 (81.1%) were currently married, 808 (68.7%) could not read and write, 1110 (94.3%) were farmers, and 393 (33.4%) were classified as economically poor (Table 1).

**Table 1:** Sociodemographic characteristics of participants in northwest Ethiopia, June-October 2020.

Variables	Frequency	Percent (%)
Sex		
Male	537	45.6
Female	640	54.4
Age, in years		
18–24	56	4.8
25–34	294	25.0
35–44	280	23.8
45–54	237	20.1
55–64	144	12.2
≥65	166	14.1
Religion		
Orthodox	1167	99.2
Muslim	10	0.8
Marital status		
Single	87	7.4
Married	955	81.1
Divorced	46	3.9
Widowed	89	7.6
Educational status		
Unable to read and write	808	68.7
Able to read and write	200	17.0
Primary school	113	9.6
Secondary and high school	42	3.5
College/University completed	14	1.2
Occupational status		
Farmer	1110	94.3
Merchant	7	0.6
Student	41	0.5
Daily laborer	5	0.4
Others	14	1.2
Wealth index*		
Poor	393	33.4
Medium	392	33.3
Rich	392	33.3

*Income is categorized based on percentiles

### Participants’ life style and behavioral characteristics

Only 4 (0.3%) and 10 (0.6%) of the total participants had ever smoked or chewed chat, respectively. One thousand one hundred twelve (94.5%) of the participants have a history of alcohol consumption, with 1073 (96.5%) having consumed alcohol in the previous 12 months. Among those who drank alcohol during the last 12 months, 360 (33.5%) and 340 (31.7%) drank 1–2 days per week and 1–3 days per month, respectively. All of the study participants consumed grain products as part of their diet. However, 989 (84.0%), 748 (63.5%), and 661 (56.2%) of study participants had never consumed fruits, vegetables, or fat-free food in the six months preceding data collection, respectively. All participants used salt when cooking food, with, 211 (17.9%) used much and too much amount of salt. Among the total participants, 740 (62.9%) were classified as having a high level of physical activity, 146 (12.4%) as low, and 1034 (87.9%) had adequate night time sleep.

### Co-morbidities and family history of hypertension related characteristics of participants

Of the participants, 128 (10.9%) had family history of hypertension (FHH). Moreover, 44 (3.7%), 22 (1.9%), 14 (1.2%), and 4 (0.3%) of the study participants had self-reported CVDs, chronic respiratory diseases, chronic kidney diseases, and diabetes mellitus, respectively. Thirty two of the participants had their blood cholesterol levels checked, with only five being diagnosed with high blood cholesterol (Table 2).

**Table 2:** Co-morbidities and FHH related characteristics of participants in northwest Ethiopia, June-October 2020.

Variables	Frequency	Percent (%)
Family history of HTN		
Yes	128	10.9
No	1049	89.1
Cardiovascular disease		
Yes	44	3.7
No	1133	96.3
Chronic respiratory disease		
Yes	22	1.9
No	1155	98.1
Chronic kidney disease		
Yes	14	1.2
No	1163	98.8
Diabetes mellitus		
Yes	4	0.3
No	1173	99.7
History of Cancer		
Yes	3	0.3
No	1174	99.7
Ever been measured for blood cholesterol		
Yes	32	2.7
No	1145	97.3
High blood cholesterol(n = 32)		
Yes	5	15.6
No	27	84.4

### Blood pressure measurements

The study participants’ mean systolic blood pressure was 117.15 mmHg (SD = ±17.60), ranging from 80 mmHg to 190 mmHg. The participants’ mean diastolic blood pressure was also 72.07 mmHg (SD±11.18) ranging from 40 mmHg to 110 mmHg.

### Prevalence of hypertension

**Fig 1:**
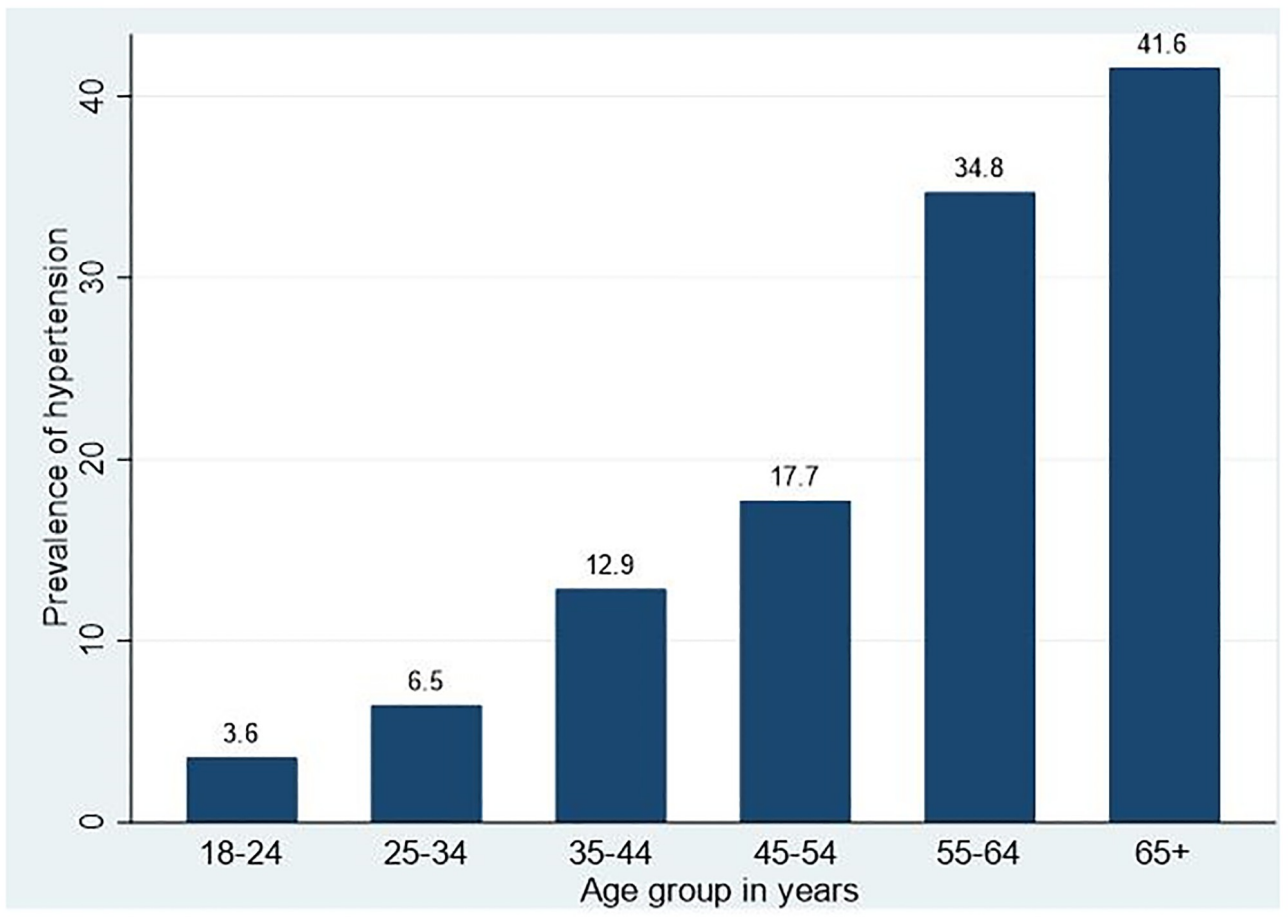
Prevalence of hypertension by age group in northwest Ethiopia, 2020.

Of the total participants, 218 (18.5%: 95% CI: 16.3, 20.7%) were found to have hypertension. The prevalence of hypertension increased consistently from 3.6% in the 18–24 age groups to 41.6% in the age group of ≥65 years (Fig 1).

Among those with hypertension, 133 (61.0%) were females, 166 (76.1%) were married, 164 (75.2%) were could not read and write. Furthermore, 213 (97.7%) hypertensive patients had ever consumed alcoholic drinks, 52 (23.9%) had a low level of physical activity, 40 (18.3%) had FHH, and 43 (19.7%) slept for less than 6 hours each day.

### Factors associated with hypertension

The bivariate logistic regression analysis revealed that being female, age≥45 years old, married, widowed, unable to read and write, having ever used alcohol, using much or too much salt, having insufficient sleep, FHH, having a low level of physical activity, and being overweight were all positively and significantly associated with hypertension. The multivariable binary logistic regression analysis, adjusted for the potential confounding variables (marital status, educational status, wealth index, level of physical activity, and BMI), being female, aged 45–54, 55–64 and ≥65 years, having ever used alcohol, using much and too much salt, having insufficient sleep, and having FHH remained positively and significantly associated with hypertension.

In this study, females were twice (AOR = 2.30, 95% CI: 1.53, 3.45) as likely to have hypertension as males. When compared to younger adults (aged 18–24 years), those aged 45–54 years were 5 times (AOR = 4.63, 95% CI: 1.01, 21.37) more likely to have hypertension, those aged 55–64 years were 14 times more likely (AOR = 14.40, 95% CI: 3.07, 67.63), and those aged ≥65 years were 19 times more likely (AOR = 19.37, 95% CI: 4.03, 93.09) to have hypertension. When compared to individuals who had never used alcohol, those who had used alcohol were 3 times (AOR = 3.25, 95% CI: 1.17, 9.02) more likely to have hypertension. Participants who used much or too much salt were 2 times (AOR = 2.37, 95% CI: 1.53, 3.60) and 4 times (AOR = 3.78, 95% CI: 1.85, 7.72) more likely to have hypertension as compared to those who used the appropriate amount of salt. Participants who slept for a short period of time were twice as likely to have hypertension as those who slept for a longer period of time (AOR = 2.05, 95%CI: 1.30, 3.24). Participants with FHH were twice (AOR = 2.12, 95%CI; 1.32, 3.39) as likely to have hypertension as compared to their counterparts (Table 3).

**Table 3:** Bivariate and multivariable logistic regression analysis of factors associated with hypertension in northwest Ethiopia, June-October 2020.

Variable	Hypertension		COR (95% CI)	AOR (95% CI)
	Yes (%)	No (%)		
Sex				
Male	85 (15.8)	452(84.2)	1	1
Female	133(20.8)	507(79.2)	1.39 (1.03, 1.88)	2.30(1.53,3.45)**
Age				
18–24	2(3.6)	54(96.4)	1	1
25–34	19(6.5)	275(93.5)	1.87(0.42, 8.24)	1.78(0.38, 8.26)
35–44	36(12.9)	244(87.1)	3.98(0.93, 17.05)	3.30(0.72, 15.24)
45–54	42(17.7)	195(82.3)	5.82(1.36, 24.80)	4.63(1.00, 21.37)*
55–64	50(34.8)	94(65.2)	14.36(3.36, 61.37)	14.40(3.07, 67.63)*
≥65	69(41.6)	97(58.4)	19.21(4.53, 81.44)	19.37(4.03, 93.09)**
Marital status				
Single	4(4.6)	83(95.4)	1	1
Married	166(17.4)	789(82.6)	4.37(1.58, 12.07)	1.86(0.62, 5.55)
Divorced	14(30.4)	32(69.6)	9.08(2.78, 29.65)	2.53(0.67, 9.60)
Widowed	34(38.2)	55(67.8)	12.83(4.31, 38.18)	1.90(0.56, 6.52)
Educational status				
Unable to read and write	164(20.3)	644(79.7)	2.14(1.27, 3.59)	0.66(0.36, 1.22)
Able to read and write	36(18.0)	164(82.0)	1.84(1.01, 3.38)	0.84(0.43, 1.65)
Primary school and above	18(10.7)	151(89.3)	1	1
Wealth index category				
Poor	86(21.9)	307(78.1)	1	1
Medium	65(16.6)	327(83.4)	0.71(0.50, 1.01)	0.96(0.63, 1.46)
Rich	67(17.1)	325(82.9)	0.74(0.52, 1.05)	0.90(0.59, 1.39)
Ever consumed alcohol				
Yes	213 (19.2)	898(80.8)	2.89 (1.15, 7.29)	3.25(1.17, 9.02)*
No	5(7.6%)	61(92.4)	1	1
Amount of salt				
Too much	21(47.7)	23(52.3)	4.98(2.66, 9.30)	3.78(1.85, 7.72)*
Much	51(30.5)	116(69.5)	2.40(1.62, 3.53)	2.37(1.53, 3.68)**
Right amount	111(15.5)	605(84.5)	1	1
Little	31(14.0)	190(86.0)	0.89(0.58, 1.37)	0.78(0.48, 1.25)
Too little	4(13.8)	25(86.2)	0.87(0.30, 2.55)	1.13(0.36, 3.53)
Sleep duration, in hours				
<6	43(30.0)	100(70.0)	2.11(1.43, 3.13)	2.05(1.30, 3.24)*
≥6	175(16.9)	859(83.1)	1	1
Family history of HTN				
Yes	40(31.3)	88(68.7)	2.22(1.48, 3.34)	2.12(1.32, 3.39)*
No	178(17.0)	871(83.0)	1	
Physical activity				
Low	52(35.6)	94(64.4)	3.24(2.18, 4.81)	1.35(0.80, 2.28)
Moderate	58(19.9)	233(80.1)	1.46(1.02, 2.07)	0.88(0.58, 1.34)
High	108(14.6)	632(85.4)	1	1
BMI				
Below normal	62(19.5)	256(80.5)	1.12(0.81, 1.57)	0.76(0.52, 1.11)
Normal	147(17.7)	685(82.3)	1	1
Overweight	9(33.3)	18(66.7)	2.33(1.03, 5.29)	1.89(0.74, 4.80)

*P-value<0.05

**P-value<0.001

Hosmer and Lemeshow Test = 0.54

## Discussion

In this study, hypertension was found to be significantly higher in the rural population, and it is emerging as a public health issue. Female sex, advanced age, ever use of alcohol, excessive salt consumption, insufficient sleep, and FHH were all positively and significantly associated with hypertension.

Hypertension is common in Ethiopia, accounting for more than half of all CVD deaths [14]. The overall prevalence of hypertension was found to be 18.5% (95% CI: 16.3, 20.7%) in this study. This finding was consistent with the findings of an Ethiopian systematic review and meta-analysis (18.45%) [21] and a study conducted in the Arba Minch Health and Demographic Surveillance Site (17.5%) [48]. It’s also similar to findings from other countries, including Ombe, Cameroon (19.8%) [49], Davanagere, India (18.3%) [50], South India (18.3%) [51], Southern Rajasthan, India (18.67%) [52], and coastal Karnataka, India (18%) [53]. The use of similar age groups of the study participants could be one reason for the similarities.

On the other hand, our finding was higher than those of studies conducted in Amhara regional state, Ethiopia (10%) [54], Southern Ethiopia (9.7%) [55], and Rural Uganda (14% [56]. The difference in time period between the current study and previous studies, which were conducted between 2008 and 2015, could be one reason for the higher prevalence of hypertension in this study. This indicates that hypertension is rising at an alarming rate among the country’s rural adult population.

Our finding, however, was much lower than those of studies conducted among rural populations in Dabat district, Northwest Ethiopia (25.3%) [24], Mali (21.1%) [57], and Nigeria (26.8%) [58]. One possible explanation is the age difference in the study population, with the median age of study participants in Dabat district (47 years) and Cameroon (53 years) being higher than in this study (40 years). This may overestimate the prevalence of hypertension, as evidenced by the older age groups showing higher prevalence [59]. The site of BP measurement (inter-arm difference) could also be a reason for the higher prevalence of hypertension in study conducted in Mali [57], where the BP was taken on the right arm of the study participants, which could result in a difference in BP reading from the left arm as high as 10 mmHg. This is supported by some studies which showed a bias towards higher readings from the right arm than the left arm [60–62].

In this study, a significant association was found between the participant’s sex and hypertension. Females were two times more likely than males to have hypertension. The possible reason might be due to the effect of hormones during menopause, which makes the BP more sensitive to salt, resulting in higher BP [63]. This result was consistent with the findings of the study conducted in Southern Rajasthan [52]. However, studies conducted in Ethiopian cities such as Gondar [26], Mekelle [64], Addis Ababa [31, 33, 34], Durame [30], Hosanna [28], and Jigjiga [65] revealed that males were at risk for hypertension.

Age is a non-modifiable risk factor for hypertension, with the risk of developing high blood pressure increasing with age [66]. The age of the participants was found to be significantly associated with the risk of hypertension in this study. Individuals aged 45–54 years, 55–64 years, and ≥65 years were 4.63, 14.40, and 19.37 times more likely to have hypertension, respectively, than those aged 18–24 years. This was consistent with studies conducted in Amhara regional state, Ethiopia [54], Dabat district and Gondar city [24], Gondar City [25, 26]. This could be because as people age, their vascular systems become less elastic, causing blood vessels to stiffen and become less compliant, raising their BP [66].

Moderate to heavy alcohol consumption significantly increases the risk of developing hypertension. In this study, participants who had ever used alcohol were 3.25 times more likely to develop hypertension than those who had never used alcohol. This corresponded to findings from studies in Dabat HDSS [67], Dabat district and Gondar town [24], Gondar city [26], Addis Ababa [33], and a systematic review and meta-analysis conducted in Ethiopia [21], which revealed that people who drank alcohol were more hypertensive than those who did not. One possible explanation is that alcohol contains calories, which can contribute to unwanted weight gain, which is a known risk factor for hypertension [68]. Second, alcohol consumption may raise BP by either inhibiting endothelial nitric oxide syntheses or inducing inflammatory injury to the endothelium, which reduces vasodilators in the vascular endothelium [69]. The third possible explanation for the association between excess alcohol consumption and high blood pressure is renin-angiotensin-aldosterone system stimulation, increased sympathetic activity, vasoconstriction, and elevated oxidative stress [69–71].

Excessive dietary salt consumption is associated with an increased risk of hypertension, which is a major risk factor for stroke, CVDs, and kidney diseases [36]. This study found that those participants who used much or too much salt were 2.37 and 3. 78 times more likely to have hypertension than those who used the right amount of salt. Studies in Durame town [30], South India [51], Korea [72], and a systematic review [73] all supported this finding, indicating that people who consumed much amounts of salt were more likely to develop hypertension than their counterparts. The pathophysiological effect of salt/sodium on blood volume explains why: increased salt consumption causes water retention and increased blood volume, which leads to a condition of high flow in arterial blood vessels. Another possible explanation is that a high sodium intake damages endothelial function, causes changes in the structure and function of large elastic arteries, and modifies sympathetic activity, resulting in peripheral vascular resistance and increases in BP [74].

Several studies have suggested that sleep duration may play an important role in the development of hypertension [75, 76]. Participants in this study who slept <6 hours per 24 hours (with short sleep duration) were twice as likely to develop hypertension as those who slept 6 or more hours per night. This was consistent with the Sleep Heart Health Study (SHHS) [47], a study conducted in northeast China among adults aged 18–44 years [77], and a meta-analysis [78–82]. This could be because short sleep durations or sleep deprivation cause over activity of the sympathetic nervous system, which leads to high blood pressure [83].

A family history of hypertension was found to be a significant factor associated with hypertension. Participants with FHH were 2.12 times more likely to have hypertension as compared to their counterparts. Studies in Amhara regional state, Ethiopia [54], Gondar city [25], Dabat district and Gondar town [24], Addis Ababa [32], Sidama Zone [55], Jimma University Specialized Hospital, Southwest Ethiopia [84], Durame town [30], Assosa town [85], Jigjiga city [65], Cameroon [86], Uganda [56], South India [51], and Sri Lankan adults [87] all supported this finding. One possible explanation is that blood relatives share many of the same genes that can predispose a person to hypertension, heart disease and stroke [88].

This study assessed the magnitude of hypertension and its associated risk factors in rural districts of Northwest Ethiopia, where the data is scarce. The prevalence of hypertension was found to be high in the rural districts of northwest Ethiopia, indicating the need for community based intervention, and the findings will be eye-opening for decision makers, implementers, healthcare providers, partners, and researchers. It also uses a standardized tool developed by WHO. However, it was also limited by the tools used, such as the assessment of behavioral risk factors using an interview technique, which could be prone to recall bias. The study did not address biochemical measurements such as blood lipid and blood glucose levels, which are major risk factors for hypertension. Furthermore, due to the use of multistage sampling, the statistical methods used may have an effect on both point and interval estimation.

## Conclusions

The finding of this study showed that hypertension is prevalent among adults in rural parts of northwest Ethiopia. Females, people over the age of 55years, people who have ever used alcohol, used much or too much salt, people who sleep for a short duration, and people who have FHH are more likely to have hypertension. Hence, we recommend that local health authorities integrate measures such as hypertension health education, lifestyle modification, including salt and alcohol reduction, and early detection of hypertension particularly among females and the elderly in rural communities.

## Supporting information

S1 Data This is the data set of the study. (DTA)

## Abbreviations

AOR: Adjusted Odds Ratio
BP: Blood Pressure
CVD: Cardiovascular Diseases
IQR: Interquartile Range
WHO: World Health Organization

## References

[1] High Blood Pressure. In: High Blood Pressure Symptoms and Causes. Centere for Disease Control and Prevention.

[2] Non-communicable diseases news. In.: World Health Organization; Pan American Health Organization; 2016.

[3] Hypertension. In.: World Health Organization; 13 September 2019.

[4] Cham B, Scholes S, Fat LN, Groce N, Mindell J: The burden of hypertension and its associated factors in the Gambia: data from a national health examination survey using the world health organisation stepwise approach. *BMJ* 2017.

[5] Yoruk A, Boulos PK, Bisognano JD: The State of Hypertension in Sub-Saharan Africa: Review and Commentary. *American Journal of Hypertension* 2018, 31(4):387–388. doi: 10.1093/ajh/hpx196 29136102

[6] Ezzati M, Vander Hoorn S, Lawes CM, Leach R, James WPT, Lopez AD, et al: Rethinking the “diseases of affluence” paradigm: global patterns of nutritional risks in relation to economic development. *PLoS medicine* 2005, 2(5):e133. doi: 10.1371/journal.pmed.0020133 15916467 PMC1088287

[7] Knott C, Mindell J: Hypertension:The health and social care information centre. *HSE* 2011, 1.

[8] WHO: For the prevention and control of Noncommunicable diseases GLOBAL ACTION PLAN 2013–2020.

[9] Okoro RN, Ngong CK: Assessment of patient’s antihypertensive medication adherence level in non-comorbid hypertension in a tertiary hospital in Nigeria. *Pharm Biomed Sci* 2012, 3(2):47–54.

[10] Chockalingam A: Impact of world hypertension day. *Canadian Journal of Cardiology* 2007, 23(7):517–519. doi: 10.1016/s0828-282x(07)70795-x 17534457 PMC2650754

[11] Alcocer L, Cueto L: Hypertension, a health economics perspective. Therapeutic advances in cardiovascular disease 2008, 2(3):147–155. doi: 10.1177/1753944708090572 19124418

[12] NONCOMMUNICABLE DISEASES PROGRESS MONITOR In. Switzerland: World Health Organization; 2017.

[13] ADDRESSING THE IMPACT OF NONCOMMUNICABLE DISEASES AND INJURIES IN ETHIOPIA: A Collaboration with the Global Lancet Commission on Reframing NCDIs for the Poorest Billion. In: Ethiopia NCDI Commission Report. Addis Ababa; November 2018.

[14] Tefera YG, Abegaz TM, Abebe TB, Mekuria AB: The changing trend of cardiovascular disease and its clinical characteristics in Ethiopia: hospital-based observational study. Vascular health risk management 2017, 13:143. doi: 10.2147/VHRM.S131259 28461753 PMC5407597

[15] Erkabu SG, Agedie Y, Mihretu DD, Semere A, Alemu YM: Ischemic and Hemorrhagic Stroke in Bahir Dar, Ethiopia: A Retrospective Hospital-Based Study. Journal of Stroke 2018, 27(6):1533–1538. doi: 10.1016/j.jstrokecerebrovasdis.2017.12.050 29397313

[16] Deresse B, Shaweno D: Epidemiology and in-hospital outcome of stroke in South Ethiopia. *Journal of the neurological sciences* 2015, 355(1–2):138–142. doi: 10.1016/j.jns.2015.06.001 26059446

[17] Temesgen TG, Teshome B, Njogu P: Treatment Outcomes and Associated Factors among Hospitalized Stroke Patients at Shashemene Referral Hospital, Ethiopia. Stroke research treatment 2018, 2018. doi: 10.1155/2018/8079578 30228857 PMC6136470

[18] Gedefa B, Menna T, Berhe T, Abera H: Assessment of Risk Factors and Treatment Outcome of Stroke Admissions at St. Paul’s Teaching Hospital, Addis Ababa, Ethiopia. *Journal of Neurology Neurophysiology* 2017, 8(03).

[19] Alemayehu CM, Birhanesilasie SK: Assessment of stoke patients: occurrence of unusually high number of haemorrhagic stroke cases in Tikur Anbessa Specialized Hospital, Addis Ababa, Ethiopia. Clinical Medicine Research 2013, 2(5):94–100.

[20] Health and Health Related Indicators. In. Addis Ababa, Ethiopia: Federal Ministry of Health; 2010.

[21] Tiruneh SA, Bukayaw YA, Yigizaw ST, Angaw DA: Prevalence of hypertension and its determinants in Ethiopia: A systematic review and meta-analysis. *Plos one* 2020, 15(12):e0244642. doi: 10.1371/journal.pone.0244642 33382819 PMC7774863

[22] Anteneh ZA, Yalew WA, Abitew DB: Prevalence and correlation of hypertension among adult population in Bahir Dar city, northwest Ethiopia: a community based cross-sectional study. International journal of general medicine 2015, 8:175. doi: 10.2147/IJGM.S81513 26005357 PMC4427605

[23] Belachew A, Tewabe T, Miskir Y, Melese E, Wubet E, Alemu S, et al: Prevalence and associated factors of hypertension among adult patients in Felege-Hiwot Comprehensive Referral Hospitals, northwest, Ethiopia: a cross-sectional study. BMC research notes 2018, 11(1):1–6. 30526686 10.1186/s13104-018-3986-1 PMC6288930

[24] Abebe SM, Berhane Y, Worku A, Getachew A: Prevalence and associated factors of hypertension: a crossectional community based study in Northwest Ethiopia. *Plos ONE* 2015, 10(4):e0125210. doi: 10.1371/journal.pone.0125210 25909382 PMC4409323

[25] Awoke A, Awoke T, Alemu S, Megabiaw B: Prevalence and associated factors of hypertension among adults in Gondar, Northwest Ethiopia: a community based cross-sectional study. *BMC cardiovascular disorders* 2012, 12(1):113. doi: 10.1186/1471-2261-12-113 23186560 PMC3519757

[26] Demisse AG, Greffie ES, Abebe SM, Bulti AB, Alemu S, Abebe B, et al: High burden of hypertension across the age groups among residents of Gondar city in Ethiopia: a population based cross sectional study. *BMC public health* 2017, 17(1):647. doi: 10.1186/s12889-017-4646-4 28793889 PMC5551023

[27] Esaiyas A, Teshome T, Kassa D: Prevalence of Hypertension and Associate Risk Factors among Workers at Hawassa University, Ethiopia: An Institution Based Cross Sectional Study. Journal of Vascular Medicine & Surgery 2018, 6(354):2.

[28] Asfaw LS, Ayanto SY, Gurmamo FL: Hypertension and its associated factors in Hosanna town, Southern Ethiopia: community based cross-sectional study. BMC research notes 2018, 11(1):306. doi: 10.1186/s13104-018-3435-1 29769149 PMC5956548

[29] Kebede B, Ayele G, Haftu D, Gebremichael G: The Prevalence and Associated Factors of Hypertension among Adults in Southern Ethiopia. *International journal of chronic diseases* 2020, 2020. doi: 10.1155/2020/8020129 32328504 PMC7171655

[30] Helelo TP, Gelaw YA, Adane AA: Prevalence and associated factors of hypertension among adults in Durame Town, Southern Ethiopia. PloS one 2014, 9(11):e112790. doi: 10.1371/journal.pone.0112790 25415321 PMC4240541

[31] Abdissa SG, Feleke Y, Awol M: Prevalence of hypertension and pre-hypertension in Addis Ababa, Ethiopia: A survey done in recognition of World Hypertension Day, 2014. The Ethiopian Journal of Health Development (EJHD) 2015, 29(1).

[32] Angaw K, Dadi AF, Alene KA: Prevalence of hypertension among federal ministry civil servants in Addis Ababa, Ethiopia: a call for a workplace-screening program. *BMC cardiovascular disorders* 2015, 15(1):76. 26197712 10.1186/s12872-015-0062-9 PMC4511244

[33] Bekele G, Tadesse T, Negaw R, Zewde T: Magnitude and associated factors of hypertension in Addis Ababa public health facilities, Ethiopia. MOJ Public Health 2018, 7(6):280–286.

[34] Asemu MM, Yalew AW, Kabeta ND, Mekonnen D: Prevalence and risk factors of hypertension among adults:A community based study in Addis Ababa, Ethiopia. PLos One 2021, 16(4). doi: 10.1371/journal.pone.0248934 33793641 PMC8016337

[35] Dahl LK: Possible role of chronic excess salt consumption in the pathogenesis of essential hypertension. *The American journal of cardiology* 1961, 8(4):571–575. doi: 10.1016/0002-9149(61)90137-0 13883095

[36] Rust P, Ekmekcioglu C: Impact of salt intake on the pathogenesis and treatment of hypertension. *Hypertension*: *from basic research to clinical practice* 2016:61–84. 10.1007/5584_2016_147 27757935

[37] He FJ, Campbell NR, Macgregor GA: Reducing salt intake to prevent hypertension and cardiovascular disease. *Revista Panamericana de Salud Pública* 2012, 32:293–300. doi: 10.1590/s1020-49892012001000008 23299291

[38] Teshome M: Agricultural Susceptibility to Climate Change in Varied Ecological areas of Northwest Ethiopia. 2017.

[39] Teshome DF, Balcha SA, Ayele TA, Atnafu A, Sisay M, Asfaw MG, et al: Trained health extension workers correctly identify high blood pressure in rural districts of northwest Ethiopia: a diagnostic accuracy study. *BMC Health Services Research* 2022, 22(1):1–9. 35317798 10.1186/s12913-022-07794-w PMC8941748

[40] Kibret KT, Mesfin YM: Prevalence of hypertension in Ethiopia: a systematic meta-analysis. *Public Health Reviews* 2015, 36(1):14. doi: 10.1186/s40985-015-0014-z 29450042 PMC5804492

[41] The WHO STEPwise approach to chronic disease risk factor surveillance (STEPS). In. Geneva, Switzerland: World Health Organization; 26 January 2017.

[42] Surveillance of chronic disease risk factors: country level data and comparable estimates. In.: World Health Organization; 2005.

[43] Ethiopia Demographic and Health Survey 2016. In. Addis Ababa, Ethiopia: Central Statistical Agency; July 2017

[44] Zegeye D, Dagnaw WW, Diro E, Feyissa YM, Azmera YM, Emyu S, et al: Ethiopian primary health care clinical guidelines. In: *Care of Children 5–14 years and Adults 15 years or older in Health Centers*. Addis Ababa: Federal Democratic Republic of Ethiopia-Ministry of Health; 2017.

[45] Global physical activity questionnaire (GPAQ) analysis guide. In. Geneva: World Health Organization; 2012: 1–22.

[46] Hwang H-R, Lee J-G, Lee S, Cha KS, Choi JH, Jeong D-W, et al: The relationship between hypertension and sleep duration: an analysis of the fifth Korea National Health and Nutrition Examination Survey (KNHANES V-3). Clinical hypertension 2015, 21(1):1–7. 26893920 10.1186/s40885-015-0020-y PMC4750796

[47] Gottlieb DJ, Redline S, Nieto FJ, Baldwin CM, Newman AB, Resnick HE, et al: Association of usual sleep duration with hypertension: the Sleep Heart Health Study. *Sleep* 2006, 29(8):1009–1014. doi: 10.1093/sleep/29.8.1009 16944668

[48] Chuka A, Gutema B, Ayele G, Megersa ND, Melketsedik ZA, Zewdie TH: Prevalence of hypertension and associated factors among adul tresidents in Arba Minch Health and Demographic Surveillance Site, Southern Ethiopia. PLos One 2020, 15(8). 10.1371/journal.pone.0237333 PMC7416932 32776993

[49] Princewel F, Cumber SN, Kimbi JA, Nkfusai CN, Keka EI, Viyoff VZ, et al: Prevalence and risk factors associated with hypertension among adults in a rural setting: the case of Ombe, Cameroon. The Pan African Medical Journal 2019, 34. 10.11604/pamj.2019.34.147.17518 PMC7025826 32117515

[50] Yuvaraj B, Nagendra Gowda M, Umakantha A: Prevalence, awareness, treatment, and control of hypertension in rural areas of Davanagere. *Indian journal of community medicine* 2010, 35(1):138. doi: 10.4103/0970-0218.62578 20606939 PMC2888343

[51] Ismail IM, Kulkarni AG, Meundi AD, Amruth M: A community-based comparative study of prevalence and risk factors of hypertension among urban and rural populations in a coastal town of South India. *Sifa Medical Journal* 2016, 3(2):41.

[52] Galav A, Bhatanagar R, Meghwal S, Jain M: Prevalence of hypertension among rural and urban population in Southern Rajasthan. *Indian Journal of Community Medicine* 2015, 6(2):41–45.

[53] Saxena T, RK H.: Prevalence of hypertension in a rural community of coastal Karnataka: a cross sectional study. *International Journal of Community Medicine and Public Health* 2017, 4(8):2774–2777.

[54] Tesfaye TD, Temesgen WA, Kasa AS, Yismaw YS: Prevalence and associated factors of hypertension in Amhara regional state city and its’ surrounding rural districts: a community-based cross-sectional study. *African health sciences* 2019, 19(3):2580–2590. doi: 10.4314/ahs.v19i3.34 32127831 PMC7040263

[55] Giday A, Tadesse B: Prevalence and determinants of hypertension in rural and urban areas of southern Ethiopia. *Ethiopian medical journal* 2011, 49(2):139–147. 21796914

[56] Kotwani P, Kwarisiima D, Clark TD, Kabami J, Geng EH, Jain V, et al: Epidemiology and awareness of hypertension in a rural Ugandan community: a cross-sectional study. *BMC Public Health* 2013, 13:1151. doi: 10.1186/1471-2458-13-1151 24321133 PMC3890617

[57] Bâ HO, Camara Y, Menta I, Sangaré I, Sidibé N, Diall I, et al: Hypertension and associated factors in rural and urban areas Mali: Data from the step 2013 survey. International journal of hypertension 2018, 2018. doi: 10.1155/2018/6959165 29610681 PMC5828104

[58] Wada O, Olawade D, Afolalu T, Oluwatofarati A, Akinwalere I: Prevalence of Hypertension among Rural Adults and Availability of Management Services in Abimbola Com-munity, Ayedaade Local Government Area, Osun State, Nigeria. Hypertension Management 2020, 6:046.

[59] Buford TW: Hypertension and aging. *Ageing research reviews* 2016, 26:96–111. doi: 10.1016/j.arr.2016.01.007 26835847 PMC4768730

[60] Cassidy P, Jones K: A study of inter-arm blood pressure differences in primary care. *Journal of human hypertension* 2001, 15(8):519–522. doi: 10.1038/sj.jhh.1001224 11494088

[61] Lane D, Beevers M, Barnes N, Bourne J, John A, Malins S, et al: Inter-arm differences in blood pressure: when are they clinically significant? *Journal of hypertension* 2002, 20(6):1089–1095. doi: 10.1097/00004872-200206000-00019 12023677

[62] Song BM, Kim HC, Shim J-S, Kang DR: Comparison between right and left upper arms in detection of hypertension. *Korean circulation journal* 2019, 49(3):267–277. doi: 10.4070/kcj.2018.0147 30468034 PMC6393325

[63] Muiesan ML, Salvetti M, Rosei CA, Paini A: Gender differences in antihypertensive treatment: myths or legends? *High blood pressure cardiovascular prevention* 2016, 23(2):105–113. doi: 10.1007/s40292-016-0148-1 27106810

[64] Bayray A, Meles KG, Sibhatu Y: Magnitude and risk factors for hypertension among public servants in Tigray, Ethiopia: A cross-sectional study. PloS one 2018, 13(10):e0204879. doi: 10.1371/journal.pone.0204879 30281660 PMC6169912

[65] Asresahegn H, Tadesse F, Beyene E: Prevalence and associated factors of hypertension among adults in Ethiopia: a community based cross-sectional study. *BMC research notes* 2017, 10(1):629. doi: 10.1186/s13104-017-2966-1 29183367 PMC5704552

[66] Pinto E: Blood pressure and ageing. *Postgraduate medical journal* 2007, 83(976):109–114. doi: 10.1136/pgmj.2006.048371 17308214 PMC2805932

[67] Abebe SM, Andargie G, Shimeka A, Alemu K, Kebede Y, Wubeshet M, et al: The prevalence of non-communicable diseases in northwest Ethiopia: survey of Dabat Health and Demographic Surveillance System. *BMJ open* 2017, 7(10):e015496. doi: 10.1136/bmjopen-2016-015496 29061601 PMC5665308

[68] Alcohol: Does it affect blood pressure? [https://www.mayoclinic.org/diseases-conditions/high-blood-pressure/expert-answers/blood-pressure/faq-20058254].

[69] Husain K, Ansari RA, Ferder L: Alcohol-induced hypertension: Mechanism and prevention. *World journal of cardiology* 2014, 6(5):245. doi: 10.4330/wjc.v6.i5.245 24891935 PMC4038773

[70] Puddey IB, Mori TA, Barden AE, Beilin LJ: Alcohol and hypertension—New insights and lingering controversies. *Current hypertension reports* 2019, 21(10):1–10. doi: 10.1007/s11906-019-0984-1 31494743

[71] Marchi KC, Muniz JJ, Tirapelli CR: Hypertension and chronic ethanol consumption: What do we know after a century of study? World journal of cardiology 2014, 6(5):283. doi: 10.4330/wjc.v6.i5.283 24944758 PMC4062120

[72] Park J, Kwock CK: Sodium intake and prevalence of hypertension, coronary heart disease, and stroke in Korean adults. Journal of Ethnic Foods 2015, 2(3):92–96.

[73] Malta D, Petersen KS, Johnson C, Trieu K, Rae S, Jefferson K, et al: High sodium intake increases blood pressure and risk of kidney disease. From the Science of Salt: A regularly updated systematic review of salt and health outcomes (August 2016 to March 2017). The Journal of Clinical Hypertension 2018, 20(12):1654–1665. doi: 10.1111/jch.13408 30402970 PMC8030856

[74] Grillo A, Salvi L, Coruzzi P, Salvi P, Parati G: Sodium intake and hypertension. *Nutrients* 2019, 11(9):1970. doi: 10.3390/nu11091970 31438636 PMC6770596

[75] Gangwisch JE, Heymsfield SB, Boden-Albala B, Buijs RM, Kreier F, Pickering TG, et al: Short sleep duration as a risk factor for hypertension: analyses of the first National Health and Nutrition Examination Survey. *hypertension Research* 2006, 47(5):833–839. doi: 10.1161/01.HYP.0000217362.34748.e0 16585410

[76] Gangwisch JE: A review of evidence for the link between sleep duration and hypertension. *American journal of hypertension* 2014, 27(10):1235–1242. doi: 10.1093/ajh/hpu071 24778107 PMC4229731

[77] Li M, Yan S, Jiang S, Ma X, Gao T, Li B: Relationship between sleep duration and hypertension in northeast China: a cross-sectional study. *BMJ open* 2019, 9(1):e023916. doi: 10.1136/bmjopen-2018-023916 30670514 PMC6347883

[78] Wang Q, Xi B, Liu M, Zhang Y, Fu M: Short sleep duration is associated with hypertension risk among adults: a systematic review and meta-analysis. *Hypertension Research* 2012, 35(10):1012–1018. doi: 10.1038/hr.2012.91 22763475

[79] Wang Y, Mei H, Jiang Y-R, Sun W-Q, Song Y-J, Liu S-J, et al: Relationship between duration of sleep and hypertension in adults: a meta-analysis. *Journal of Clinical Sleep Medicine* 2015, 11(9):1047–1056. doi: 10.5664/jcsm.5024 25902823 PMC4543249

[80] Guo X, Zheng L, Wang J, Zhang X, Zhang X, Li J, et al: Epidemiological evidence for the link between sleep duration and high blood pressure: a systematic review and meta-analysis. *Sleep medicine* 2013, 14(4):324–332. doi: 10.1016/j.sleep.2012.12.001 23394772

[81] Meng L, Zheng Y, Hui R: The relationship of sleep duration and insomnia to risk of hypertension incidence: a meta-analysis of prospective cohort studies. *Hypertension Research* 2013, 36(11):985–995. doi: 10.1038/hr.2013.70 24005775 PMC3819519

[82] Santos EdSGd, Souza OFd: Evidence of the association between sleep duration and blood pressure in adolescents: a systematic review. *Revista Paulista de Pediatria* 2020, 39. doi: 10.1590/1984-0462/2021/39/2019225 32785432 PMC7409100

[83] Tochikubo O, Ikeda A, Miyajima E, Ishii M: Effects of insufficient sleep on blood pressure monitored by a new multibiomedical recorder. *Hypertension Research* 1996, 27(6):1318–1324. doi: 10.1161/01.hyp.27.6.1318 8641742

[84] Gudina EK, Michael Y, Assegid S: Prevalence of hypertension and its risk factors in southwest Ethiopia: a hospital-based cross-sectional survey. *Integrated blood pressure control* 2013, 6:111. doi: 10.2147/IBPC.S47298 23986649 PMC3753877

[85] Gadisa LM: Prevalence and Associated Factors of Hypertension among Assosa Town, Western-Ethiopia, 2018. Clinics in Medicine 2020, 2(2).

[86] Simo LP, Agbor VN, Noubiap JJN, Nana OP, Nkosu PS-M, Anouboweh AFA, et al: Hypertension prevalence, associated factors, treatment and control in rural Cameroon: a cross-sectional study. BMJ open 2020, 10(9):e040981. doi: 10.1136/bmjopen-2020-040981 32907908 PMC7482484

[87] Ranasinghe P, Cooray DN, Jayawardena R, Katulanda P: The influence of family history of hypertension on disease prevalence and associated metabolic risk factors among Sri Lankan adults. *BMC public health* 2015, 15(1):1–9. 26092387 10.1186/s12889-015-1927-7 PMC4475303

[88] Peng Q, Shao Y-q, Fang X, Zhang Y-y: The interaction on hypertension between family history and diabetes and other risk factors. *Scientific Reports* 2021, 11(1):1–7. 33633182 10.1038/s41598-021-83589-z PMC7907071

